# The cytosolic form of dual localized BolA family protein Bol3 is important for adaptation to iron starvation in *Aspergillus fumigatus*

**DOI:** 10.1098/rsob.240033

**Published:** 2024-06-26

**Authors:** Simon Oberegger, Matthias Misslinger, Klaus Faserl, Bettina Sarg, Hesso Farhan, Hubertus Haas

**Affiliations:** ^1^ Institute of Molecular Biology, Biocenter, Medical University Innsbruck, Innsbruck, Austria; ^2^ Institute of Medical Biochemistry, Biocenter, Medical University Innsbruck, Innsbruck, Austria; ^3^ Institute of Pathophysiology, Biocenter, Medical University Innsbruck, Innsbruck, Austria

**Keywords:** filamentous fungi, mould, *Aspergillus fumigatus*, iron, iron–sulfur cluster, chaperone

## Abstract

*Aspergillus fumigatus* is the predominant mould pathogen for humans. Adaption to host-imposed iron limitation has previously been demonstrated to be essential for its virulence. [2Fe–2S] clusters are crucial as cofactors of several metabolic pathways and mediate cytosolic/nuclear iron sensing in fungi including *A. fumigatus*. [2Fe–2S] cluster trafficking has been shown to involve BolA family proteins in both mitochondria and the cytosol/nucleus. Interestingly, both *A. fumigatus* homologues, termed Bol1 and Bol3, possess mitochondrial targeting sequences, suggesting the lack of cytosolic/nuclear versions. Here, we show by the combination of mutational, proteomic and fluorescence microscopic analyses that expression of the Bol3 encoding gene leads to dual localization of gene products to mitochondria and the cytosol/nucleus via alternative translation initiation downstream of the mitochondrial targeting sequence, which appears to be highly conserved in various *Aspergillus* species. Lack of either mitochondrial Bol1 or Bol3 was phenotypically inconspicuous while lack of cytosolic/nuclear Bol3 impaired growth during iron limitation but not iron sensing which indicates a particular importance of [2Fe–2S] cluster trafficking during iron limitation. Remarkably, cytosolic/nuclear Bol3 differs from the mitochondrial version only by N-terminal acetylation, a finding that was only possible by mutational hypothesis testing.

## 1. Introduction

*Aspergillus fumigatus* is one of the most ubiquitous airborne saprobic fungi. In addition, this mould is an opportunistic pathogen that can cause various diseases, including invasive aspergillosis, which is associated with high mortality rates, particularly in immunocompromised patients [1]. Its virulence depends on adaptation to the host niche including efficient uptake of trace elements. Iron is an essential trace element for all eukaryotes but toxic in excess [2]. Moreover, this metal plays a key role in host–pathogen interaction. Pathogens are usually confronted with limited iron availability within the mammalian host owing to ‘nutritional immunity’, which can lead to ‘anaemia of inflammation’ during infection [3,4]. Consequently, pathogens evolved strategies to adapt to iron limitation including high-affinity iron acquisition mechanisms and respective regulatory circuits ensuring sufficient iron supply but preventing iron toxicity. *A. fumigatus* is a prime example for the importance of iron homeostasis in virulence. In this mould, siderophore-mediated iron acquisition, as well as transcriptional iron regulation have been demonstrated to be crucial for pathogenicity [5–8]. In *A. fumigatus* iron homeostasis is mainly regulated by two iron-responsive transcription factors (TFs), termed HapX and SreA. HapX but not SreA, was found to be crucial for virulence of *A. fumigatus* [7,9]. During iron starvation, HapX represses genes involved in iron-consuming pathways and, upon a shift to iron excess, HapX activates the same genes [7,10]. SreA blocks the expression of genes needed for iron uptake during sufficient iron availability [9]. Several studies indicated that these two TFs sense the cellular iron status via iron–sulfur (FeS) cluster binding [11–13].

FeS clusters play diverse and indispensable roles as cofactors in various cellular processes including cellular respiration, nucleotide biosynthesis and repair, as well as biosynthesis of amino acids, proteins and vitamins [14–16]. The most common FeS clusters are the rhombic [2Fe–2S] and the cubane [4Fe–4S] clusters [15]. The biosynthetic pathway for these FeS clusters can be roughly subdivided into three parts localized in two cellular compartments: the mitochondrial iron–sulfur cluster machinery (ISC), consisting of [2Fe–2S] cluster biosynthesis (core ISC), the build-on mitochondrial [4Fe–4S] cluster biosynthesis machinery (late ISC) and the cytosolic [4Fe–4S] cluster assembly (CIA), which depends on an exported product of the core ISC [17,18]. FeS cluster biosynthesis has been studied in greatest detail in *Saccharomyces cerevisiae* and appears to be highly conserved within eukaryotes [18]. Trafficking of FeS clusters to client proteins requires chaperones that can reversibly bind FeS clusters. The best understood [2Fe–2S] cluster trafficking proteins are monothiol glutaredoxins (mGrx), also termed class II glutaredoxins or CGFS glutaredoxins, and BolA proteins [19]. mGrx coordinates [2Fe–2S] cluster in a homodimeric complex, including additionally two glutathione molecules, or can form [2Fe–2S]-bridged heterocomplexes with BolA proteins. All analysed eukaryotes possess mGrx and BolA versions in both mitochondria and cytosol. The mitochondrial versions are imported into mitochondria by mitochondrial targeting sequences (MTS). For example, *S. cerevisiae*, *Schizosaccharomyces pombe* and *A. fumigatus* possess a single mitochondrial mGrx; *S. pombe* and *A. fumigatus* possess also a single cytosolic/nuclear mGrx, while *S. cerevisiae* employs two paralogues in this compartment [19]. Both *S. pombe* and *S. cerevisiae* possess one cytosolic/nuclear and two mitochondrial BolA versions; the mitochondrial versions are termed Bol1 and Bol3 and the cytosolic version Bol2 or Fra2 [19]. Interestingly, both homologues of *A. fumigatus* and other *Aspergillus* species, termed Bol1 and Bol3, possess putative MTS [12], suggesting the lack of cytosolic/nuclear versions, which would be unique among eukaryotes.

Several lines of evidence indicate that *A. fumigatus* HapX and SreA sense the availability of cellular iron by binding of [2Fe2S] clusters: (i) correct transcriptional iron response was found to depend on the core mitochondrial [2Fe–2S] cluster synthesizing ISC but not on the cytosolic [4Fe–4S] cluster synthesizing CIA [11]; (ii) mutation of putative [2Fe–2S] clusters coordinating amino acids impaired iron sensing [10]; (iii) a recombinant HapX protein displayed a UV–visible spectrum indicative of the presence of [2Fe–2S] clusters; and (iv) the single cytosolic mGrx homologue GrxD was found to interact with both HapX and SreA for removal of [2Fe–2S] clusters to mediate adaptation to iron limitation [12]. Similarly, iron regulatory TFs were shown to sense iron by binding [2Fe–2S] clusters in *S. cerevisiae* and *S. pombe* but in these yeast species cytosolic mGrx was shown to be required also for trafficking [2Fe–2S] clusters to the TFs, i.e. adaptation to iron availability [20–22]. Moreover, in these two yeast species, iron sensing was shown to involve [2Fe–2S]-bridged heterocomplexes of mGrx with BolA proteins, termed Fra2 in *S. pombe* and formerly Fra2 and now Bol2 in *S. cerevisiae* [23,24].

The goal of the present study was to elucidate if *A. fumigatus* indeed lacks a cytosolic BolA protein and to investigate the role of a potential cytosolic BolA protein in iron homeostasis of this fungal pathogen.

## 2. Results

### 2.1. *In silico* analysis indicates that *bol3* might encode dual localized proteins

**Figure 1.**
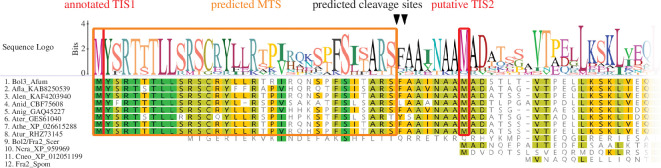
Alignment of Bol3 homologues of different *Aspergillus* species and MTS-lacking Bol2/Fra2 homologues from *S. cerevisiae*, *S. pombe*, *N. crassa* and *C. neoformans*. The alignment shows only the 60 N-terminal amino acids; the complete alignment is shown in electronic supplementary material, figure S2. The putative MTS is boxed in orange and the predicted cleavage sites are indicated by black triangles. A downstream methionine residue (Met41), which is perfectly conserved in the Bol3 homologues from other *Aspergillus* species and aligns to the N-termini of Bol2 homologues from *N. crassa* (Ncra) and *C. neoformans* (Cneo) that lack MTS, is highlighted by a red box (putative TIS2). Further aligned species: *A. fumigatus* (Afum), *A. flavus* (Afla), *A. lentulus* (Alen), *A. nidulans* (Anid), *A. niger* (Anig), *A. terreus* (Ater), *A. thermomutans* (Athe), *A. turcosus* (Atur), *S. cerevisiae* (Scer) and *S. pombe* (Spom).

*A. fumigatus* was reported to possess two BolA proteins, termed Bol1 and Bol3, which was confirmed by blastp and tblastn homology searches (https://blast.ncbi.nlm.nih.gov/Blast.cgi) using all six BolA protein sequences from *S. cerevisiae* and *S. pombe*, termed Bol1, Bol2/Fra2 and Bol3 [19]. As reported previously [12] and supported by bioinformatical analysis using MitoFates [25], both homologues are predicted to possess N-terminal MTS (scores of 0.993 for Bol3 and 0.997 for Bol1), which indicated the lack of cytosolic/nuclear BolA versions in *A. fumigatus*. In agreement, *A. fumigatus* Bol1 was previously shown to localize to mitochondria [12]. Deletion of the gene encoding Bol1 in *A. fumigatus* AfS77, termed wild-type (wt) here, by replacement with the hygromycin resistance cassette (*hph*) did not cause any phenotypic changes under different growth conditions (electronic supplementary material, figure S1). Therefore, we focused on further analyses of Bol3. An alignment of the N-terminal of Bol3 homologues illustrated that the MTS is conserved in all analysed *Aspergillus* species (figure 1). In contrast, *Neurospora crassa* and *Cryptococcus neoformans* possess BolA homologues lacking MTS similar to *S. cerevisiae* Bol2 and *S. pombe* Bol2/Fra2 (figure 1). The alignment of the full-length sequences can be found in electronic supplementary material, figure S2.

After translocation into the mitochondrial matrix, MTS are cleaved off and the cleavage site of Bol3 predicted by MitoFates [25] is RSF↓AA (↓ shows the cleavage site; figure 1), which is in perfect agreement with the consensus sequence RX (F/Y/L)↓(A/S)X (/ indicates alternative amino acid residues within parentheses) indicating initial cleavage by the mitochondrial processing peptidase at RS↓FAA followed by cleavage of the N-terminal Phe residue by ICP55 [26–29]. As displayed in figure 1, the cleavage site is highly conserved in other *Aspergillus* species. Taken together, this analysis indicated that Bol3 is a mitochondrial localized protein and that mitochondrial maturation results in cleavage of the N-terminal 34 amino acid residues and Ala35 being the N-terminal amino acid residue of the processed Bol3 protein. Together with the previously demonstrated mitochondrial localization of Bol1 [12], these data suggested that *A. fumigatus* and other *Aspergillus* species lack a cytosolic BolA protein, which would contrast with other eukaryotic organisms [19].

Interestingly, the alignment of Bol3 homologues from different *Aspergillus* species revealed a conserved methionine residue downstream of the predicted MTS cleavage site, Met41, which roughly corresponds to the N-terminal of MTS-lacking Bol2 proteins from *N. crassa* and *C. neoformans* (figure 1). Therefore, we hypothesized that the corresponding AUG codon of Met41 serves as an alternative translation initiation site (TIS), TIS2, enabling the production of a cytosolic/nuclear version (Bol3c) besides the mitochondrial form (Bol3m) originating from TIS1.

### 2.2. Lack of *bol3* causes a growth defect, which is largely rescued by high iron supplementation

**Figure 2.**
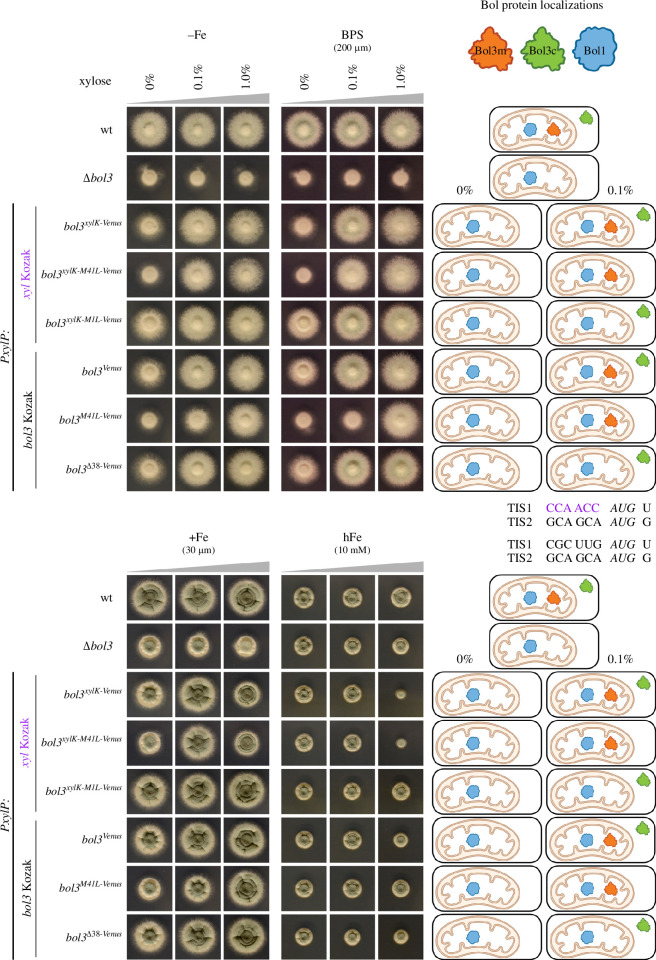
Loss of either *bol3* (*Δbol3*) or Bol3c causes a growth defect under iron limitation (−Fe and BPS), while overexpression of Bol3m causes a growth defect under iron excess (hFe). 10^4^ spores of respective strains were point-inoculated onto minimal media reflecting iron limitation (−Fe and BPS), moderate iron supply (+Fe) and iron excess (hFe). The *bol3* alleles under *PxylP* control were either non-induced (0% xylose) or induced by supplementation with 0.1 and 1.0% xylose, respectively. Plates were incubated at 37°C for 48 h. Electronic supplementary material, figure S3, shows the hFe condition after 72 h of incubation. Subcellular localization of Bol1 as well as predicted localization of Bol3m and Bol3c are shown in the schemes on the right-hand side. The introduced artificial *xylP* Kozak sequence is marked in purple.

To functionally analyse Bol3, the encoding gene was deleted in wt by replacement with the pyrithiamine (*ptrA*) resistance marker gene, resulting in strain *Δbol3*. Owing to a possible role in iron homeostasis, growth assays were carried out on minimal media reflecting different iron availability (figure 2), i.e. iron limitation (−Fe), iron limitation with addition of the ferrous iron-specific chelator bathophenanthrolinedisulfonic acid (BPS; 0.001 mM iron plus 0.2 mM BPS), moderate iron availability (+Fe; 0.03 mM iron) and iron excess (hFe, 10 mM iron). The *Δbol3* strain displayed a severe growth defect under iron limiting conditions (−Fe and BPS) and moderate iron supply (+Fe), but not under iron excess (hFe). These results indicate that Bol3 plays a role in maintaining iron homeostasis in *A. fumigatus*, particularly under iron limitation.

To ensure monitoring of gene deletion-specific effects, *bol3* was reintegrated into *Δbol3*, however, under the control of the *xylP* promoter (*PxylP*), which allows conditional and tunable gene expression dependent on the level of xylose supplementation [30,31], and C-terminally tagged with the coding region for the yellow fluorescent protein Venus to allow monitoring of Bol3 at the protein level [32]. Western blot analysis of this strain, termed *bol3*^Venus^, demonstrated that production of Bol3 is not detectable under non-inducing conditions (without xylose addition), clearly detectable in the presence of 0.1% xylose and further increased about 3-fold in the presence of 1% xylose (electronic supplementary material, figure S4). According to our hypothesis, *bol3* contains two TIS, termed TIS1 and TIS2 (figure 1). Downstream alternative translational initiation (TI) occurs usually during leaky scanning, i.e. if the initial TIS is weak [33]. The selection and strength of a TIS are determined by the context of the translation start codon, termed Kozak sequence. Therefore, *bol3^Venus^* contained the original six nucleotides (5′-CGCUUG) upstream of TIS1 (figure 2). With moderate induction (0.1% xylose), the *bol3^Venus^* strain displayed largely wt-like growth under all growth conditions tested (figure 2). With high induction (1% xylose), *bol3^Venus^* also showed wt-like growth with the exception of slightly reduced growth under iron excess. The latter indicates that *bol3* overexpression is detrimental under iron excess. Without induction (0% xylose), *bol3^Venus^* showed a growth defect under all conditions except under iron excess. Consequently, the growth pattern of *bol3^Venus^* was similar to that of *Δbol3*, but the growth defect was less severe, which can most likely be explained by the basal *PxylP* activity in *bol3^Venus^*. As *PxylP* shows low basal activity [31,34], these data indicate that low *bol3* expression already impacts the growth pattern. The *Δbol3* strain displayed a similar growth defect also on complex medium (electronic supplementary material, figure S5), revealing that the growth defect cannot be cured by supplementation with metabolites present in yeast extract or peptone such as amino acids or vitamins. Taken together, these data indicate that lack of *bol3* causes an iron availability-dependent growth defect and that the used complementation allele is fully functional.

### 2.3. Lack of putative Bol3c but not of putative Bol3m causes an iron availability-dependent growth defect and overexpression of putative Bol3m but not of putative Bol3c is detrimental during iron excess

**Figure 3.**
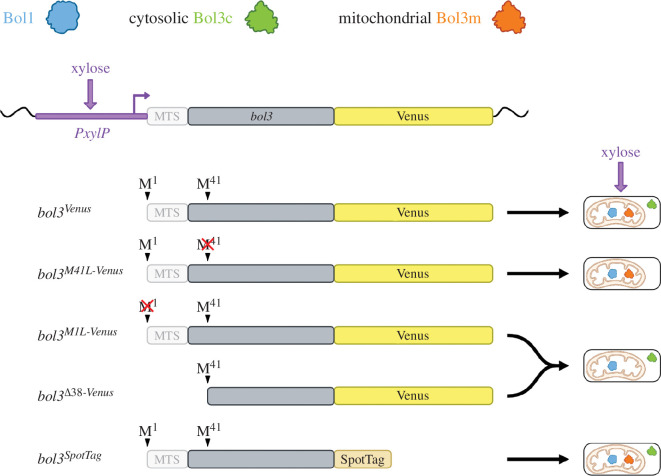
The schematic overview of the analysed *bol3* alleles. The *bol3* coding sequence excluding the MTS is shown in grey, the N-terminal MTS in white and the C-terminally fused Venus in yellow. All versions were expressed under the control of the xylose-inducible *PxylP* shown in purple. In *bol3^M41L-Venus^*, putative TIS2 was inactivated by mutation of Met41 (AUG) into Leu (CTT) to eliminate putative cytosolic Bol3c. In *bol3^M1L-Venus^*, TIS1 was inactivated by mutation of Met1 (AUG) into Leu (CTT) to eliminate mitochondrial Bol3m. In *bol3^Δ38-Venus^*, mitochondrial Bol3m was eliminated by deletion of the region encoding the first 38 amino acid residues including the MTS and the six-nucleotide-long Kozak sequence of TIS1. In front of TIS1, the genes contained either the endogenous *bol3* Kozak sequence (*bol3^Venus^* and *bol3^M41L-Venus^*) or that of *PxylP* (*bol3^xylK-Venus^*, *bol3^xylK-M41L-Venus^* and *bol3^xylK-M1L-Venus^*).

To probe our hypothesis of two alternative TIS in *bol3* (figure 1), we generated several mutant strains depicted in figure 3. Elimination of putative Bol3m by deletion of the region encoding the first 38 amino acid residues including the MTS, resulting in strain *bol3^Δ38-Venus^*, caused a growth pattern largely identical to that of *bol3^Venus^* under all tested conditions (figure 2). These data indicate that lack of putative Bol3m is not responsible for the growth defect caused by deletion of *bol3* (*Δbol3*). To eliminate putative Bol3c, we inactivated the putative TIS2 by exchange of methionine at position 41 by leucine (AUG to CTT), resulting in strain *bol3^M41L-Venus^* (figure 3). Compared with *bol3^Venus^*, *bol3^M41L-Venus^* displayed decreased growth with 0% and 0.1% xylose under all conditions but iron excess (figure 2). These data indicate that the growth defect of *Δbol3* is likely based on the lack of putative Bol3c. Interestingly, high expression with 1% xylose cured the growth defect under all conditions and under iron excess *bol3^M41L-Venus^* displayed even better growth than *bol3^Venus^*. Why should the defect caused by the loss of Bol3c be cured by the overproduction of Bol3m? It has been reported previously that exceeding the capacities of mitochondrial import and protein processing leads to the accumulation and erroneous localization of mitochondrially targeted proteins in the cytosol [28]. Therefore, the loss of Bol3c might be compensated by wrongly localized Bol3m during overexpression. Alternatively, poor recognition of TIS2 might be sufficient to produce enough Bol3c to cure the growth defect under overexpression conditions.

Downstream alternative TI usually requires leaky scanning of the first TIS, which depends on the sequence context, termed Kozak sequence. To probe this, we exchanged the Kozak sequence of TIS1 (5′-CGCUUG) by the Kozak sequence of the *PxylP*-driven gene (5′-CCAACC) (figure 2), which is assumed to mediate strong TI [31,35]. Exchange of the Kozak sequence combined with an exchange of methionine at position 1 by leucine (exchange of AUG to CTT; figure 3) to inactivate the assumed TIS1 leading to strain *bol3^xylK-M1L-Venus^* did not have phenotypical consequences, which is in agreement with dispensability of Bol3m. Interestingly, the exchange of the Kozak sequence in *bol3^Venus^*, leading to strain *bol3^xylK-Venus^*, which is assumed to increase Bol3m and to eliminate or at least decrease Bol3c did not cause significant changes in the growth phenotypes. Moreover, the exchange of the Kozak sequence in *bol3^M41L-Venus^* leading to strain *bol3^xylK-M41L-Venus^* decreased the growth defect under iron limiting conditions (−Fe and BPS) with moderate induction (figure 2; 0.1% xylose). As this strain lacks putative Bol3c, these results appear to contradict the hypothesis of Bol3c being responsible for the growth defect. However, vast overexpression of Bol3m owing to stronger TI might compensate the loss of Bol3c by cytosolic localization owing to saturation of the mitochondrial protein import machinery as discussed above for overexpression of *bol3^M41L-Venus^* via increased transcription with 1% xylose [28]. Interestingly, overexpression with 1% xylose of *bol3^xylK-M41L-Venus^* and *bol3^xylK-Venus^* caused a growth defect compared to *bol3^-M41L-Venus^* and *bol3^Venus^*, respectively, on moderate iron supply (+Fe) and particularly iron excess (hFe). These data indicate that overexpression of Bol3m is detrimental during iron excess. In line, *bol3^xylK-M1L-Venus^*, which is assumed to lack Bol3m, does not show this effect.

**Figure 4.**
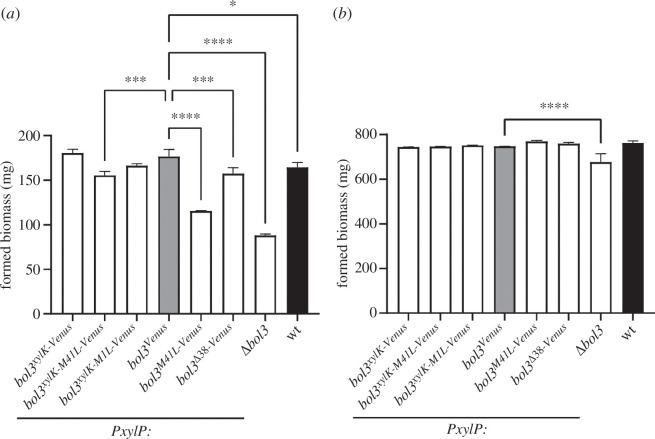
Strains *Δbol3* and *bol3^M41L-Venus^* display an iron availability-dependent growth defect in liquid shake flask culture. Fungal strains were grown under iron limitation (−Fe; *a*) and moderate iron supply (+Fe; *b*) with 0.1% xylose supplementation for *PxylP* induction for 24 h at 37°C with 200 r.p.m. shaking. The values shown represent the means ± s.d. of the dry masses of biological triplicates. Levels of significance, calculated by applying a two-way analysis of variance (ANOVA) in comparison to strain *bol3^Venus^*, are indicated by asterisks (****p < 0.0001; ***p < 0.001; *p < 0.05).

In order to obtain sufficient biomass for biochemical analyses, it is necessary to grow the fungal strains in liquid shake flask cultures. Therefore, we analysed the growth pattern of all described strains in liquid shake flask cultures with 0.1% xylose induction (figure 4). Similar to the plate growth assays, *Δbol3* and *bol3^M41L-Venus^* displayed the most severe growth defect under iron limitation (−Fe; figure 4*a*). Furthermore, exchange of the original Kozak sequence by that of *xylP* as well as moderate iron supply (+Fe) improved growth of *Δbol3* and *bol3^M41L-Venus^*. Consequently, all strains showed similar biomass formation under moderate iron supply with the exception of a mild growth defect of *Δbol3* (figure 4*b*).

### 2.4. Proteomic analyses confirmed that expression of *bol3* leads to proteins with different N-termini and consequently different localization

**Figure 5.**
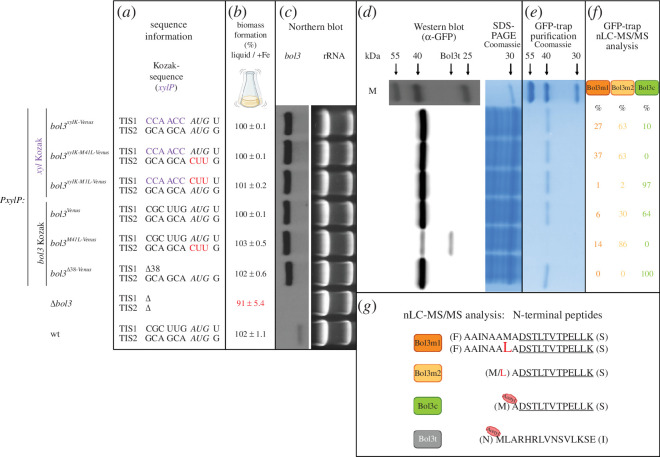
The nLC-MS/MS analysis confirmed the existence of Bol3 protein variants with different cellular localization, most likely mitochondrial and cytosolic. The indicated strains with displayed Kozak sequences of initial TIS1 and alternative TIS2 (*a*) were subject to determination of biomass formation in liquid culture (*b*) described in figure 4, northern blot analysis (*c*), western blot analysis (*d*), GFP-trap purification of Venus-tagged proteins followed by SDS-PAGE analysis (*e*) and nLC-MS/MS analysis of trypsin-digested proteins from GFP-trap enrichment. (*a*) The putative start codons are highlighted in italics; introduced mutations of the start codons are shown in red; the introduced *xylP* Kozak sequence is shown in purple; (*c*) Ethidium bromide stained ribosomal RNA (rRNA) is shown as control for quality and quantitiy of the used total RNA; (*d*) a protein ladder (M) is included for determination of the molecular mass and Coomassie-stained SDS-polyacrylamide gel after electrophoretic separation of protein extracts is included as loading control for the protein extracts; (*e*) a protein ladder (M) is included for determination of the molecular mass; (*f*) the table shows semiquantitative analysis of the ratios of the peptides representing the N-terminal of Bol3m1, Bol3m2 and Bol3c within each strain; (*g*) amino acid residues within parentheses are flanking the peptide in the protein but are not present in the peptide; the leucine residue originating from inactivation of TIS2 by the Met41Leu exchange is marked in red, and identical amino acid residues in Bol3m1, Bol3m2 and Bol3c are underlined.

The dry masses of the fungal strains cultivated under moderate iron supply were subject to northern blot analysis, western blot analysis, GFP-trap purification followed by SDS-PAGE analysis and mass spectrometry analysis (figure 5).

Northern blot analysis revealed similar *bol3* transcript levels in all strains expressing *bol3* under *PxylP* control (figure 5*c*). These transcript levels were significantly higher compared to wt, indicating that 0.1% xylose induction already leads to overexpression of *bol3*, and the transcripts were larger in size which agrees with the fusion with the Venus-coding region. The slightly shorter transcript in strain *bol3^Δ38-Venus^* is consistent with the deletion of 114 nt in this transcript. Moreover, the northern blot analysis confirmed the lack of a *bol3* transcript in *Δbol3* (figure 5*c*).

Western blot analysis of cell extracts (figure 5*d*), using an α-GFP antibody for detection of Venus-tagged Bol3 proteins, revealed a single protein with a size of approximately 38 kDa, which roughly matches the size of putative Bol3c (10.2 kDa) plus that of Venus (27.2 kDa; including the linker sequence) yielding 37.4 kDa, in similar amounts in all strains with one exception. The expected molecular mass of Bol3m (after cleavage of the predicted MTS) differs only by six amino acid residues (corresponding to 0.5 kDa) from Bol3c. Consequently, putative Bol3c and Bol3m might not be distinguishable in this analysis. Notably, strain *bol3^M41L-Venus^* showed a significantly lower protein amount of the 38 kDa protein. The latter might be explained by low amounts of Bol3m owing to the expected ‘weak’ TIS1, as in *bol3^Venus^*, combined with lack of Bol3c, in contrast to *bol3^Venus^*. According to this assumption, the majority of the 38 kDa Venus-tagged protein fraction in strains with the original Kozak sequence consists of Bol3c. Moreover, strain *bol3^M41L-Venus^* displayed an additional protein, termed Bol3t (t for truncated) with a size of about 32 kDa, which is slightly larger than Venus. In the described approach, discrimination of putative Bol3c and Bol3m might have been difficult owing to the large size of the Venus tag. Therefore, we generated another strain, *bol3^SpotTag^*, expressing *bol3* under *PxylP* control with the original Kozak sequence and C-terminally tagged with the small SpotTag (1.6 kDa). Nevertheless, western blot analysis of this strain did not reveal the presence of Bol3 proteins of different sizes (electronic supplementary material, figure S6). Taken together, western blot analysis did not discriminate proteins of different sizes but demonstrated a decrease of *bol3*-encoded protein amount upon inactivation of putative TIS2 by the Met41Leu exchange (strain *bol3^M41L-Venus^*).

In the next step, total cell extracts were subjected to GFP-trap purification for enrichment of Venus-tagged proteins [36]. SDS-PAGE followed by Coomassie staining reproduced the western blot results (figure 5*e*): similar amounts of *bol3*-encoded proteins were enriched from all strains but *bol3^M41L-Venus^*. The latter strain showed a significantly lower amount of the 38 kDa protein and the smaller Bol3t protein, which could hardly be detected.

Next, GFP-trap enriched Bol3 protein fractions from the different mutant strains were subject to nano-liquid chromatography–tandem mass spectrometry (nLC-MS/MS) analyses after digestion with trypsin, which preferentially cleaves at the C-terminal side of either lysine or arginine residues. This analysis revealed four major peptides (figure 5*g*), revealing the presence of *bol3*-encoded proteins showing differences in the N-terminus: [M]A*DSTLTVTPELLK[S], [F]AAINAAMADSTLTVTPELLK[S], [F]AAINAALADSTLTVTPELLK[S] and [M/L]ADSTLTVTPELLK[S] (* indicates N-terminal acetylation of alanine; amino acid residues within brackets are flanking the peptide in the protein but are not present in the peptide; identical amino acid residues are underlined; the leucine residue originating from inactivation of TIS2 by the Met41Leu exchange is marked in red). The nLC-MS/MS results are shown in electronic supplementary material, table S1. These four peptides most likely represent the N-terminal of Bol3 variants as (i) all four peptides contain a C-terminal lysine owing to processing by trypsin but lack an N-terminal lysine indicating its origin either by TI or proteolytic processing without action of trypsin; (ii) all four peptides contain the same C-terminal 12 amino acid residues but differ in N-terminal amino acid residues; and (iii) all peptides cover the predicted N-terminal of Bol3m and Bol3c (figure 1).

There are several lines of evidence that the peptide [M]A*DSTLTVTPELLK[S] represents the N-terminus of Bol3c, i.e. it is indicative for the use of TIS2 and consequently alternative TI downstream of the MTS-encoding region followed by loss of the N-terminal methionine residue and acetylation of the new N-terminal alanine residue (figure 5*g*). First, this peptide was present in all strains with the exception of *bol3^M41L-Venus^* and *bol3^xylK-M41L-Venus^* (figure 5*f*), which were expected to lack Bol3c owing to mutation of TIS2 leading to the Met41Leu exchange. Second, this peptide lacks the predicted translation start methionine and shows N-terminal acetylation of the amino acid residue following methionine, which takes place in a co- or post-translational manner (see §3). N-terminal methionine cleavage and alanine acetylation appear to be highly efficient as almost 100% of the N-terminal Bol3c peptides were found to possess this modification in strains *bol3^Δ38-Venus^* and *bol3^xylK-M1L-Venus^*, which are predicted to lack Bol3m (figure 5*f*,*g*; electronic supplementary material, table S1).

The other three peptides, [F]AAINAAMADSTLTVTPELLK[S], [F]AAINAALADSTLTVTPELLK[S] and [M/L]ADSTLTVTPELLK[S], are indicative for the utilization of TIS1 and consequently Bol3m (figure 5*g*). In agreement, all three peptides were absent in *bol3^Δ38-Venus^* and largely missing in *bol3^xylK-M1L-Venus^*, which lack putative Bol3m owing to lack of the MTS or mutation of TIS1 (figures 3 and 5*f*), respectively. The first two peptides were identical with the exception of the Met41Leu exchange. In line, [F]AAINAAMADSTLTVTPELLK[S] was found exclusively in *bol3^Venus^* and *bol3^xylK-Venus^* and [F]AAINAALADSTLTVTPELLK[S] only in *bol3^M41L-Venus^* and *bol3^xylK-M41L-Venus^* (electronic supplementary material, table S1). These two peptides contain the N-terminus predicted by processing of the MTS within the mitochondrial matrix (figure 1) and, therefore, the respective protein was termed Bol3m1. Interestingly, these peptides were found in significantly lower amounts compared to [M/L]ADSTLTVTPELLK[S], i.e. about 17% in *bol3^Venus^*, 14% in *bol3^M41L-Venus^*, 30% in *bol3^xylK-Venus^* and 37% in *bol3 ^xylK-M41L-Venus^* (figure 5*f* and electronic supplementary material, table S1). We termed the respective protein Bol3m2. Notably, the detected Bol3m2 peptide is N-terminal seven amino acid residues shorter than the Bol3m1 N-terminal peptides (figure 5*g*). Consequently, Bol3m2 is most likely generated by further N-terminal processing of Bol3m1. Remarkably, these analyses revealed that the most abundant mitochondrial Bol3 version, Bol3m2, is identical to cytosolic Bol3c with the exception of N-terminal acetylation of Bol3c (figure 5*f*,*g*). These results are in agreement with western blot and GFP-trap analyses, which did not reveal *bol3*-encoded proteins that differ in size (see §2.4). The nLC-MS/MS analyses also revealed a significant impact of the Kozak sequence on TI (figure 5*f*). In *bol3^Venus^*, containing the original Kozak sequence, 64% of the *bol3*-encoded proteins were cytosolic Bol3c. This decreased to 10% in *bol3^xylK-Venus^*, which contains the strong *xylP* Kozak sequence for the mitochondrial Bol3m proteins. These results are in agreement with efficient alternative downstream TI being dependent on leaky upstream scanning. Also, the ratio of differently processed mitochondrial Bol3m1 and Bol3m2 was affected by the Kozak sequence. In the strains containing the original Kozak sequence, *bol3^Venus^* and *bol3^M41L-Venus^*, Bol3m1 made up 17% and 14% of the mitochondrial proteins, respectively (figure 5*f* and electronic supplementary material, table S1). Exchange by the strong *xylP* Kozak sequence in corresponding strains *bol3^xylK-Venus^* and *bol3 ^xylK-M41L-Venus^* increased this ratio to 30 and 37%, respectively. This indicates that overexpression of Bol3m saturates the machinery for processing of N-terminal within mitochondria.

Western blot and GFP-trap analyses described above indicated a fourth protein, termed Bol3t, found exclusively in strain *bol3^M41L-Venus^*, which contained highly decreased amounts of Bol3 proteins (figure 5*d*,*e*). Its detection by α-GFP antibody indicated that it contains Venus. As its size was slightly larger than that of the Venus tag, we hypothesized its N-terminus within the C-terminus of Bol3. The nLC-MS/MS analyses described above could not identify the N-terminus of this protein. However, the expected region displays an unfavourable distribution of lysine and arginine residues for detection of peptides using trypsin digestion. Applying digestion with endoproteinase Glu-C (V8), which cleaves preferentially at the C-terminal side of either glutamic or aspartic acid residues, revealed a peptide in *bol3^M41L-Venus^* that originates from the C-terminal region of Bol3 (Met95; electronic supplementary material, figure S2), most likely representing the Bol3t N-terminus, [N]M*LARHRLVNSVLKSE[I] (figure 5*g* and electronic supplementary material, table S2). This peptide also displayed N-terminal acetylation, typical for cytosolic TI; the lack of methionine removal is consistent with the following amino acid residue being a bulky leucine residue [37,38]. These results indicate that weak recognition of TIS1 in *bol3 ^M41L-Venus^*, combined with mutation of TIS2, leads to further scanning and recognition of an artificial TIS causing the production of Bol3t. In agreement with the weak or lacking recognition of the initial TIS1 and the mutated alternative TIS2, only *bol3 ^M41L-Venus^* displayed highly reduced amounts of the 38 kDa Bol3 proteins in western blot and GFP-trap analyses (figure 5*d*,*e*). These results are in perfect agreement with the ribosomal scanning mechanism for TI.

### 2.5. Fluorescence microscopy confirms dual localization of Bol3 in *A. fumigatus*

**Figure 6.**
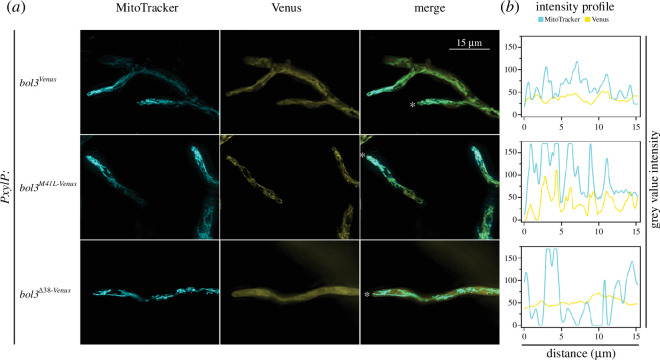
Fluorescence microscopy confirms the dual localization of Bol3 in strain *bol3^Venus^* and its exclusive localization in the cytosol or mitochondria in mutant strains *bol3^Δ38-Venus^* and *bol3^M41L-Venus^*, respectively. (*a*) Pictures are representative images for the strains grown under moderate iron supply with 0.1% xylose supplementation for 16 h at 37°C. Venus-derived fluorescence is shown in yellow. Mitochondrial staining using MitoTracker Deep Red FM is shown in blue. Asterisks (*) at hyphae tips in the merged pictures indicate the starting point of the 15 µm longitudinal intensity profile measurements shown in (*b*).

In the next step, the subcellular localization of Venus-tagged Bol3 versions was analysed by fluorescence microscopy in mutant strains *bol3^Venus^*, *bol3^M41L-Venus^* and *bol3^Δ38-Venus^*, which contain the original Kozak sequence (figure 6*a*). According to the nLC-MS/MS analyses (figure 5*f*), *bol3^Venus^* produces both mitochondrial and the cytosolic Bol3 versions, while *bol3^M41L-Venus^* and *bol3^Δ38-Venus^* lack the cytosolic or the mitochondrial versions, respectively. Mitochondria were visualized by staining with MitoTracker™ Deep Red FM.

Fluorescence imaging (figure 6*a*) and intensity profiles (figure 6*b*) of Venus- and MitoTracker-derived fluorescence illustrated homogeneous cytosolic distribution of Venus-derived fluorescence without correlation with MitoTracker-derived fluorescence in *bol3^Δ38-Venus^*, which is in agreement with the predominant production of Bol3c and the lack of mitochondrial Bol3m1 and Bol3m2. In contrast, *bol3^M41L-Venus^* displayed exclusive mitochondrial localization of Venus-derived fluorescence strongly correlating with MitoTracker-derived fluorescence, which is consistent with the predominant production of mitochondrial Bol3 versions and the lack of Bol3c in this strain. Strain *bol3^Venus^* displayed an intermediate state compared to the two strains described. In strain *bol3^Venus^*, Venus-derived fluorescence was clearly cytosolically localized but additionally correlated with mitochondria, which is in agreement with the production of both mitochondrial and cytosolic Bol3 versions. Notably, nLC-MS/MS analysis indicated that about 64% of the Bol3 proteins are cytosolic Bol3c (figure 5*f*), which explains the rather weak correlation of Venus- and MitoTracker-derived fluorescence intensity profiles compared to strain *bol3^M41L-Venus^* (figure 6*b*). Taken together, fluorescence microscopy of the three mutant strains confirmed dual localization of Bol3, predicted by bioinformatic analysis, growth phenotyping and particularly nLC-MS/MS analyses.

### 2.6. Bol3 proteins do not play a major role in iron sensing in *A. fumigatus*

**Figure 7.**
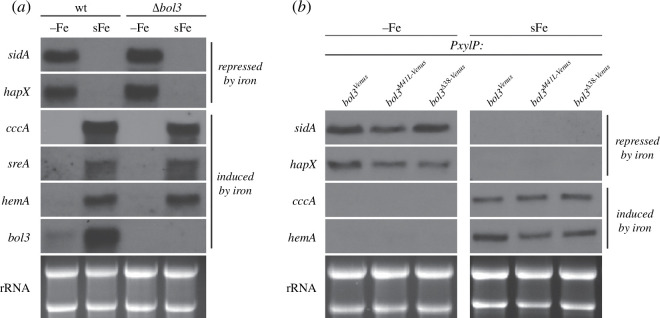
Lack of neither *bol3* (*Δbol3*; *a*) nor cytosolic/nuclear (*bol3^M41L-Venus^*) or mitochondrial (*bol3^Δ38-Venus^*) Bol3 versions (*b*) significantly impacts iron regulation in *A. fumigatus*. For northern blot analysis, total RNA was isolated from liquid shake flask cultures incubated at 37°C for 16 h under iron limitation (−Fe) or for 15.5 h under iron limitation followed by short-term iron exposure (sFe; 10 µM iron) for another 0.5 h for induction of iron-responsive genes. (*b*) Cultures for strains expressing *bol3* versions under *PxylP* control were supplemented with 0.1% xylose. Hybridization probes included genes that are repressed (*sidA*, *hapX*) or induced (*cccA*, *hemA*) by iron. Ethidium bromide-stained rRNA is shown as loading and quality control for used RNAs.

Several lines of evidence indicated that *A. fumigatus* iron regulatory TF HapX and SreA sense the availability of cellular iron by binding of [2Fe–2S] clusters, via CRR involving the [2Fe–2S] cluster chaperon GrxD [10–12]. To analyse whether Bol3c is involved in iron sensing in *A. fumigatus*, wt and *Δbol3* strains were cultivated under iron limitation (−Fe) as well as short-term exposure to iron (sFe) followed by northern blot analysis, targeting iron-regulated genes. The wt as well as the *Δbol3* strain showed the previously reported iron limitation response [7,9,10,12], i.e. induction siderophore biosynthetic *sidA* and iron regulatory *hapX* as well as repression of the vacuolar iron importer *cccA* and heme biosynthetic *hemA* (figure 7*a*). Moreover, in short-term iron exposure (sFe), both strains displayed the previously reported repression of *sidA* and *hapX* as well as the induction of *cccA* and *hemA* (figure 7*a*). Notably, *bol3* transcript levels were significantly upregulated under short-term iron exposure (sFe) in wt. Consistent with *bol3* gene deletion, *bol3* transcripts were not detected in *Δbol3* (figure 7*a*). In line with dispensability of the different Bol3 versions for iron regulation, individual lack of mitochondrial (*bol3^Δ38-Venus^*) or cytosolic/nuclear (*bol3^M41L-Venus^*) Bol3 versions did not impair transcriptional iron regulation (figure 7*b*).

## 3. Discussion

By the combination of mutational analysis, phenotyping, northern blot analysis, western blot analysis, proteomic analysis and fluorescence microscopic investigation (figures 2, 3, 5*d–g*, 6 and 7), we demonstrated that expression of *bol3* leads to dual localized gene products owing to alternative TI including and excluding the MTS in *A. fumigatus* and most likely other *Aspergillus* species. Phylogenetic analysis indicated that the dual localization of *bol3*-derived proteins via alternative TI is conserved only within Eurotiomycetidae including not only Eurotiales genera such as *Aspergillus* and *Penicillium* but also *Onygenales* species such as *Histoplasma capsulatum*, *Paracoccidioides brasiliensis* and *Coccidioides immitis* (data not shown). Consequently, *A. fumigatus* does not represent an exception by lacking a cytosolic BolA version, as expected from genome annotation. Interestingly, phylogenetic analysis indicated that *A. fumigatus* Bol3 is closer related to Bol2/BolA2 homologues than to Bol3/BolA3 homologues, while Bol1 is a classical Bol1/BolA1 subfamily member [39]. Consequently, *A. fumigatus* appears to lack a classical Bol3/BolA3 homologue.

Lack of *bol3* caused a growth defect under iron limitation on solid as well as in liquid media (*Δbol3*; figures 2 and 4), which was largely cured by high iron supplementation. Generation of mutants that lack either the mitochondrial (Bol3m, missing in *bol3^Δ38-Venus^* and *bol3^xylK-M1L-Venus^*) or the cytosolic/nuclear version (Bol3c, missing in *bol3^M41L-Venus^* and *bol3^xylK-M41L-Venus^*) owing to inactivation of the respective TIS or specific deletion of the MTS (figure 3) demonstrated that the growth defect is caused by lack of cytosolic Bol3c, while lack of mitochondrial Bol3m was phenotypically inconspicuous (figure 2). Similarly, lack of the other mitochondrial BolA protein, Bol1, did not cause phenotypic changes (electronic supplementary material, figure S1). Either Bol3m and Bol1 are not important for growth under the tested conditions or compensate for each other’s absence. Notably, only the combinatorial elimination of both mitochondrial BolA proteins, Bol1 and Bol3, but not their individual elimination had phenotypical consequences in *S. cerevisiae* [40].

Conditional expression of C-terminally Venus-tagged *bol3* versions (figure 3) allowed purification and subcellular localization of the expressed protein variants. Based on the detection of peptides that show N-terminal differences, nLC-MS/MS analysis revealed that expression of *bol3* leads to three different Bol3 versions (figure 5*g*): the hypothesized cytosolic Bol3c and two mitochondrial versions, termed Bol3m1 and Bol3m2. The N-terminal Bol3c peptide shows hallmarks for origin by cytosolic TI, i.e. cleavage of the start methionine residue and acetylation of the novel N-terminal alanine residue [38,41,42].

A high portion of cytosolic eukaryotic proteins is subject to removal of the N-terminal methionine and N-terminal acetylation, taking place in a co-translational or post-translational manner by ribosomal-bound methionine aminopeptidases and acetyltransferases [38,41,42]. Cleavage of the N-terminal methionine residue takes mainly place when it is followed by a small amino acid residue, e.g. Gly, Ala, Val, Ser, Cys, Pro or Thr [37,38], which is in agreement with the acetylated N-terminal alanine residue in the putative N-terminal Bol3c peptide (figure 5*g*). In contrast, N-terminal acetylation has not been reported after proteolytic cleavage apart from cleavage of methionine during TI. N-terminal acetylation of proteins influences protein properties such as protein stability, protein folding, protein–protein interactions and the subcellular targeting of proteins [43]. Depending on the organism, about 50–80% of cytosolic protein species have been reported to be N-terminally acetylated in yeast and human, respectively [43,44]. Furthermore, it has been shown that N-terminal acetylation harbours the potential to impair protein targeting to subcellular compartments [45]. In agreement with mitochondrial localization, N-terminal peptides characteristic for Bol3m1 and Bol3m2 (figure 5*g*) were absent in strains, in which TIS1 preceding the MTS was inactivated (*bol3^Δ38-Venus^* and *bol3^xylK-M1L-Venus^*). Bol3m1 displayed the N-terminus following cleavage of the MTS (figures 1 and 5*g*) and further processing in the mitochondrial matrix predicted by MitoFates (figures 1 and 5*g*) [25]. Remarkably, Bol3m2 lacked the N-terminal six amino acids compared to Bol3m1 (figures 1 and 5*g*) and had the same N-terminus as Bol3c but lacking the N-terminal acetylation (figure 5*g*). Bol3m2 is most likely derived by further processing of Bol3m1. In line, a variety of N-terminal maturation processes have been characterized [26–29]. Bol3m2 was found to make up the majority of mitochondrial Bol3 proteins (figure 5*f* and electronic supplementary material, table S1). Consequently, the major mitochondrial Bol3 version differs from the cytosolic Bol3 version only by 43 Da. In agreement, neither SDS-PAGE nor western blot analyses revealed different Bol3 versions, neither by C-terminal tagging with large Venus nor with small SpotTag (figure 5*d*; electronic supplementary material, figures S4 and S6).

Alternative TI is usually enabled by leaky scanning of the initial TIS, which is based on the sequence context, the so-called Kozak sequence. Consequently, TIS1 of *bol3* (5′-CGCUU*GAUG*U) is expected to be weak. In agreement, it significantly differs from that of the *PxylP*-driven gene (5′-CCAACC*AUG*U), which mediates strong TI [31,35]. The most preferred Kozak consensus sequences preceding the TIS in mammals and in *N. crassa* are 5′-GCC(A/G)CC and, similarly, 5′-N(C/U)CA(C/A)(C/A), respectively [33,46]. These sequences largely match that of the strong *PxylP* (5′-CCAACC) in contrast to the native, weak Kozak-sequence driving the expression of Bol3m (5′-CGCUUG) (figure 2). Moreover, the Kozak sequence of TIS2 driving expression of Bol3c shows higher resemblance with strong Kozak sequences. In line, exchange of the native Kozak sequence of TIS1 by that of the *xylP* gene (*bol3^Venus^* versus *bol3^xylK-Venus^*) increased the portion of mitochondrial Bol3 proteins compared to all Bol3 proteins from 36 to 90% (figure 5*f*), which is in agreement with efficient alternative TI being dependent on weak initial TI. Moreover, total Bol3 protein production of strains with native and *xylP* gene Kozak sequences was largely identical (*bol3^Venus^* and *bol3^xylK-Venus^*; figure 5*d*,*e* and electronic supplementary material, figure S4), which indicates that the combination of weak native TIS1 and strong TIS2 (*bol3^Venus^*) provides similar protein production to the strong TIS1 of the *xylP* gene (*bol3^xylK-Venus^*). The Kozak sequence exchange also changed the ratio of differently processed mitochondrial Bol3 versions (figure 5*f*): it increased the amount of Bol3m1 compared to further processed Bol3m2. These data indicate that overexpression of Bol3m saturates the mitochondrial machinery for N-terminal protein processing.

Strains carrying *bol3* under *PxylP* control (e.g. *bol3^Venus^* and *bol3^xylK-Venus^*) showed a growth defect under non-inducing conditions that was less severe than that caused by *bol3* deletion (*Δbol3*; figure 2). This is most likely caused by the basal *PxylP* activity. As *PxylP* shows very low basal activity [31,34], these data indicate that low *bol3* expression already affects the growth pattern, or in other words, that only low Bol3c amounts are required for its cellular function. Interestingly, overexpression of Bol3m owing to high *PxylP* induction (*bol3^M41L-Venus^* with 1% xylose; figure 2) or exchange of the Kozak sequence (*bol3^xylK-M41L-Venus^* with 0.1% xylose; figure 2) cured the growth defect caused by lack of Bol3c (*bol3^M41L-Venus^* with 0.1% xylose; figure 2). It has been reported previously that exceeding the capacities of mitochondrial import and protein processing leads to the accumulation and erroneous localization of mitochondrially targeted proteins in the cytosol [28]. Therefore, our data indicate that loss of Bol3c is compensated by wrongly localized Bol3m during overexpression wing to transcriptional upregulation or increased translation via Kozak sequence exchange. Alternatively, poor recognition of TIS2 might be sufficient to produce enough Bol3c to cure the growth defect under overexpression conditions.

Efficient adaption to iron limitation has been shown to be crucial for virulence of the human pathogen *A. fumigatus* [7]. Owing to the iron-dependent growth defect caused by the lack of Bol3c, this Bol3 version plays most likely an important role in virulence. Recent studies indicated that this mould senses the cellular iron status via cellular availability of [2Fe–2S] clusters [13]. In both *S. cerevisiae* and *S. pombe*, cytosolic mGrx and BolA proteins were found to be involved in [2Fe–2S] cluster trafficking to and from iron-regulatory TF [19]. In *A. fumigatus*, the cytosolic mGrx, GrxD, was suggested to mediate removal of [2Fe–2S] clusters from the iron regulatory TF SreA and HapX for adaptation to iron availability [12]. Moreover, this study indicated that *A. fumigatus* uses GrxD-independent mechanism for sensing iron sufficiency. Here, we demonstrate by northern blot-mediated gene expression analysis that Bol3c does not play a major role in iron sensing (figure 7). Nevertheless, lack of Bol3c caused a growth defect that was largely cured by iron supplementation (*bol3^M41L-Venus^* with 0.1% xylose; figures 2 and 4*b*), which indicates that Bol3c is particularly important for adaptation to iron limitation. Most likely these data reflect the importance of Bol3c-mediated [2Fe–2S] cluster trafficking under conditions of [2Fe–2S] cluster shortage caused by iron limitation. The functions of the cytosolic/nuclear BolA proteins from yeast species, other than their role in iron sensing, are unknown. However, several studies indicated a role of BOLA2 in mammalian cytosolic iron trafficking. BOLA2 was indicated to form an Fe(II)-bridged complex with glutathione and the cytosolic iron chaperone PCBP1, which acquires Fe(II) from the labile iron pool [47]. This complex is proposed to serve as an intermediate complex required for the assembly of [2Fe–2S] clusters on BolA2-Glrx3, thereby linking ferrous iron and FeS cluster distribution systems in cells. Moreover, BOLA2 has been shown to deliver [2Fe–2S] clusters together with the mGrx GLRX3 to CIA components in *in vitro* studies that assembles and delivers [4Fe–4S] clusters [19,48]. In humans, the gene encoding BolA2 (*BOLA2*) is located in a region displaying copy-number polymorphic duplications, which are under positive selection and include three to eight copies of *BOLA2* [49]. Analysis of phenotypes associated with *BOLA2* copy-number variation revealed reduced *BOLA2* dosage associated with mild anaemia while, inversely, increased *BOLA2* dosage improves systemic iron homeostasis. In line, mice lacking BOLA2 show early evidence of iron deficiency [49]. These results indicate that BOLA2 participates in mammalian iron homeostasis *in vivo*, and its expansion has a potential adaptive role in protecting against iron deficiency. In this regard, *A. fumigatus* matches mammals as the lack of Bol3c (*bol3^M41L-Venus^* with 0.1% xylose; figure 2) caused a growth defect during iron limitation that was largely cured by iron supplementation. In contrast, overexpression of mitochondrial Bol3m was detrimental in *A. fumigatus* during iron excess conditions (*bol3^xylK-Venus^* and *bol3^xylK-M41L-Venus^* with 1% xylose; figure 2) indicating the role of mitochondrial BolA proteins in mitochondrial iron homeostasis.

Eukaryotic protein synthesis generally initiates at a start codon defined by an AUG and its surrounding Kozak sequence context [33]. This can be wrong in two directions: (i) poor-context AUGs may be skipped or leaky scanned and (ii) non-AUGs may be also recognized [46,50]. Expression of the *bol3* gene represents a prime example of the plasticity of TI and the importance of TIS sequence context as an important regulatory signal that programs both the abundance and the structure of proteins. Dual localization wing to leaky scanning of the initial TIS followed by a strong Kozak context in-frame AUG, separated by MTS, has been reported previously in *C. neoformans* [51]. However, the cytosolic BolA protein in *C. neoformans* is encoded by an MTS-lacking gene (figure 1). Identification of the dual localization of *bol3*-encoded gene products would not have been possible using classical proteomics without the prediction of the Bol3c and Bol3m N-termini as proteomics is based on annotated protein sequences. Moreover, the finding that the major mitochondrial and cytosolic Bol3 versions of *A. fumigatus*, Bol3m2 and Bol3c, differ only in N-terminal acetylation could not have been obtained without the mutational approach used.

## 4. Material and methods

### 4.1. Growth conditions

Unless otherwise specified, *Aspergillus* minimal medium [52] containing 1% (w/v) glucose and 20 mM glutamine as carbon and nitrogen sources, respectively, was used for cultivation of *A. fumigatus* strains. For iron limited conditions, addition of iron was either omitted (−Fe) or media contained 0.001 mM FeSO_4_ plus 0.2 mM of the ferrous iron-specific chelator BPS. For moderate iron supply (+Fe) and iron excess (hFe), media contained 0.03 mM and 10 mM FeSO_4_, respectively. *Aspergillus* complex medium contained 2% (w/v) glucose, 0.2% (w/v) peptone, 0.1% (w/v) yeast extract and 0.1% (w/v) NZ-casamino acids according to [52] but without the addition of iron, unless stated otherwise. For fungal transformation and spore production, +Fe medium supplemented with 3 µM ferricrocin to facilitate germination and growth was used. Liquid shake flask cultures were inoculated with a final spore concentration of 10^6^ spores per millilitre of medium and culturing was conducted with shaking at 200 r.p.m. All incubations were performed at 37°C. Genes under the control of *PxylP* were induced by the addition of 0.1% or 1.0% (w/v) xylose.

### 4.2. Mutant strain generation

The genetic background for all strains generated in this study was *A. fumigatus* strain AfS77 (termed wt here), derived from *A. fumigatus* ATCC46645 but lacking non-homologous end joining (*ΔakuA::loxP*) [53]. All mutant strains used in this study were generated using the transformation procedure according to [54]. A summary of all primers used in this study can be found in electronic supplementary material, table S3.

Deletion of *bol1* (Afu7g01520) and *bol3* (Afu6g12490) as well as generation of the *PxylP*-driven *bol3* versions (figures 2 and 3) C-terminal tagged with Venus [32] or SpotTag (ChromoTek) is described in detail in the electronic supplementary material. These *bol3* versions were inserted at the *fcyB* locus [55] in the *∆bol3* strain.

Genetic manipulations were confirmed by sequencing of plasmids in combination with southern blot analysis of transformants described in electronic supplementary material, figure S7. All used fungal strains are summarized in electronic supplementary material, table S4.

### 4.3. Nucleic acid isolation, northern blot and southern blot analyses

Isolation of total RNA was performed using TRI reagent (Sigma-Aldrich) according to the manufacturer’s protocol. Subsequently, 10 µg of total RNA was separated on a 1.2% (w/v) agarose gel containing 1.85% (w/v) formaldehyde and then blotted onto a Hybond™-N+ membrane (Amersham Biosciences). Transcripts of interest were detected with digoxigenin-labelled (Roche) hybridization probes generated by PCR amplification.

DNA was isolated by PCI extraction and isopropanol precipitation. To confirm the gene-specific restriction pattern of the genetic manipulations, DNA was digested with restriction enzymes specific for the respective gene. The resulting restriction fragments were separated on agarose gels and transferred to Amersham™ Hybond™-N Membranes (Amersham Biosciences) by capillary blotting with NaOH. Signals for correct integration were detected using digoxigenin-labelled (Roche) probes amplified by PCR.

The primers used for preparation of northern and southern blot hybridization probes are listed in electronic supplementary material, tables S3 and S5, respectively.

### 4.4. Protein extraction and western blot analysis

Harvested biomass from liquid cultures was freeze-dried and proteins were extracted by alkaline lysis of freeze-dried mycelia, followed by protein precipitation using trichloroacetic acid [56]. After electrophoresis with 12–20% SDS-polyacrylamide gels, proteins were blotted onto a nitrocellulose membrane (Amersham™ Protran™ Premium 0.45 µm NC, GE Healthcare). Proteins of interest were detected with mouse α-GFP antibody (1:10 000 diluted; Roche) or mouse α-SpotTag antibody (1:5000 diluted; ChromoTek) as primary antibodies in combination with peroxidase-coupled secondary anti-mouse antibody (1:10 000 diluted; Sigma-Aldrich). For detection, the ECL reagent (Amersham Biosciences) was used.

### 4.5. GFP-TRAP and SPOT-CAP purification

Protein extraction and enrichment of GFP- and SpotTag-tagged proteins were performed using 20 mg freeze-dried mycelium per 500 µl lysis buffer largely according to the manufacturer’s instructions (ChromoTek). For inhibition of proteases, complete ULTRA EDTA-free (Roche) was added to the lysis buffer. GFP- and SpotTag-tagged proteins were eluted from beads using 1% (v/v) formic acid and 100 mM glycine (pH 2), respectively. For western blot analysis, samples were neutralized by titrating with 1 M Tris, pH 10.4.

### 4.6. Fluorescence microscopy

Fungal strains with an inoculum of 10^5^ spores per millilitre were grown in 8-well chamber slides (µ-Slide 8 Well; Ibidi) with a total volume of 200 µl minimal medium. Mitochondrial staining was performed for 1 h using MitoTracker™ Deep Red FM (Invitrogen™) in a final concentration of 500 nM. Images were acquired with a 60× TIRF objective (Plan-APOCHROMAT 60×/1.49 Oil, Nikon) mounted on an inverted microscope (Eclipse Ti2-E; Nikon) with a spinning disc confocal unit (CSU-W1, Yokogawa), an EMCCD camera (iXon Ultra 888, Andor) with an additional 1.5× magnification and using the NIS-Elements software (Nikon). The Lucy–Richardson algorithm was applied for deconvolution and Fiji ImageJ [57] for processing of microscopy pictures.

### 4.7. nLC-MS/MS analysis

Freeze-dried GFP-trap purified samples (figure 5*e*) were reduced with 40 µl of 10 mM dithiothreitol in ABC-buffer (100 mM ammonium bicarbonate, pH 8.0) at 56°C for 30 min. Proteins were then in-solution digested with 0.1 µg trypsin (Promega) for 6 h or 0.2 µg of chymotrypsin (Sigma-Aldrich) for 3 h or under agitation at 37°C. Free cysteines were alkylated by adding 40 µl of 55 mM iodoacetamide in ABC buffer followed by incubation at room temperature for 20 min in the dark.

Digested peptides were analysed using an UltiMate 3000 nano‐HPLC system coupled to a Q Exactive Plus mass spectrometer (Thermo Scientific) as described previously [58]. Peptides were separated on a 17 cm long column (100 μm i.d.) packed with 2.4 μm C18 material (Reprosil). Solvents for nano-HPLC were 0.1% formic acid and 0.1% formic acid in 85% acetonitrile. Total gradient time was 82 min at a flow rate of 300 nl min^−1^. The 20 most abundant peptides in the full MS scan were selected for MS fragmentation. The isolation window was set to 1.6 *m*/*z*. Full scan spectra were acquired from 300 to 1750 *m*/*z* at a resolution of 60 000. Peptides were fragmented by HCD with a normalized collision energy set to 28 and scanned at a resolution of 30 000.

The MS data files were processed using Proteome Discoverer version 2.2 (Thermo Scientific) in combination with the Sequest HT search engine. MS/MS spectra were searched against a database containing the hypothesized mature Bol3 protein versions followed by a second search against the *A. fumigatus* proteome database. Enzyme specificity was set to unspecific when searching against Bol3 protein versions and set to trypsin (two missed cleavages allowed) when searching against the *A. fumigatus* proteome database. The fixed modification was carbamidomethyl on cysteine; variable modifications were oxidation of methionine and acetylation and/or methionine loss of peptide N-terminal. Precursor mass tolerance was set to 10 ppm; fragment mass tolerance was 20 mmu. The maximum false-discovery rate for protein and peptide identification was set to 1%. For label-free quantification, the Minora Feature Detector node was set to high confidence peptide spectrum matches with a minimum of two isotopic peaks present in the isotope pattern. Retention time alignment was performed at a maximum retention time shift of 10 min and a mass tolerance of 10 ppm.

### 4.8. Database searches and statistical analyses

Nucleotide as well as protein sequences were obtained from FungiDB [59] and NCBI databases [60], whereby the latter was also used for carrying out BLAST searches. Protein alignments and sequence figures were performed using Geneious Prime (2023, v1.2) [61]. For planning and designing transformation constructs the cloud-based platform Benchling (Biology Software, 2023) was used. Prediction of putative MTS and cleavage sites was performed using MitoFates [25].

For statistical analyses, GraphPad Prism version 9.1.0 for Windows was used (GraphPad Software, www.graphpad.com).

## Data Availability

The datasets presented in this article are included in the paper or available in the electronic supplementary material [62]. Sequences were obtained from FungiDB [59]; gene numbers are mentioned in the Material and methods section and are listed in electronic supplementary material, table S5.
